# Optimal management of ANCA-associated vasculitis before and during pregnancy: current perspectives

**DOI:** 10.1007/s00404-022-06744-5

**Published:** 2022-09-14

**Authors:** Ann-Christin Pecher, Melanie Henes, Joerg Christoph Henes

**Affiliations:** 1 Centre for Interdisciplinary Clinical Immunology, Rheumatology and Autoinflammatory Diseases and Department of Internal Medicine II, University Hospital Tuebingen, Otfried-Mueller-Strasse 10, 72076 Tuebingen, Germany; 2 Department of Obstetrics and Gynaecology, University Hospital Tuebingen, Tuebingen, Germany

**Keywords:** Autoimmune, Pregnancy, ANCA, Treatment, Vasculitis

## Abstract

Antineutrophil cytoplasmic antibody (ANCA)-associated vasculitides (AAV) are a group of systemic vasculitis characterized by autoantibodies against neutrophil cytoplasmic antigens (proteinase 3 PR3-ANCA and myeloperoxidase MPO-ANCA) and inflammation of small vessels. AAV include the diagnosis *Granulomatosis with polyangiitis* (GPA), *microscopic polyangiitis* (MPA) and *eosinophilic granulomatosis with polyangiitis* (EGPA), which share many clinical and pathological features. Immunomodulatory therapies have significantly improved prognosis during the last decade. Nevertheless, especially in undiagnosed and thus uncontrolled AAV mortality due to renal impairment or pulmonary haemorrhages is still high. AAV are rare in fertile women, as the typical age of manifestation is above 50 years but there are women with AAV who are or want to become pregnant. This review focusses on how to manage patients with AAV planning to become pregnant and during their pregnancy.

## What does this study add to the clinical work

**Table:** 

Pregnancies in ANCA associated vasculitis are rare but physician need to be aware of expectable problems. With a close monitoring and interdisciplinary consultations successful pregnancy can be achieved in most of our patients.	

## Introduction

Antineutrophil cytoplasmic antibody (ANCA)-associated vasculitides (AAV) are a group of systemic vasculitis characterized by inflammation of small vessels and in most of the cases presence of autoantibodies against neutrophil cytoplasmic antigens (proteinase 3 PR3-ANCA and myeloperoxidase MPO-ANCA). AAV include the diagnosis *Granulomatosis with polyangiitis* (GPA), *microscopic polyangiitis* (MPA) and *eosinophilic granulomatosis with polyangiitis* (EGPA), which share several clinical and pathological features but also have their specificities (see Table 1). Every organ might be involved; however, the upper and lower respiratory system and kidneys are most commonly affected.

**Table 1:** Common clinical and epidemiology features of antineutrophil cytoplasmic antibody (ANCA)-associated vasculitides

	GPA	MPA	EGPA
Incidence per person-years	0.4–11.9 per million	0.5–24 per million	0.5–2.3 per million
Age of onset in years	45–65	55–75	38–54
Granulomas	Yes	No	Yes
Eosinophilia	No	No	Yes + eosinophilic infiltrations e.g. of the lung
Allergic features	No	No	Rhinitis, asthma
ENT involvement	+ + +	+	+ +
Lung involvement	+ +	+ +	+ + +
Renal involvement	+ +	+ + +	+
Nerve involvement	+	+ +	+ + +
ANCA	50–90% positive, mostly PR3-ANCA	90% positive, mostly MPO-ANCA	30–50% ANCA positive, mostly MPO-ANCA

Adapted from [1]

*GPA* granulomatosis with polyangiitis, *MPA* microscopic polyangiitis, *EGPA* eosinophilic granulomatosis with polyangiitis, *ENT* ear nose throat

AAV are rare diseases with incidence rates between 0.4 and 24 cases per million [1]. Due to the initial manifestation usually after the age of 50 [2] pregnancies in AAV are uncommon, but there are women affected during their fertile years and pregnancies occur. In addition, pregnancy might be a trigger for AAV, as there are several cases which describe the first presentation of AAV during pregnancy, with severe disease courses [3, 4]. Data from pregnant patients with AAV mostly derive from case reports or retrospective studies. Since 2015, the Vasculitis Pregnancy Registry (V-PREG) collects data on pregnant women with several different vasculitides, including 24 patients with AAV in the first 3 years [5]. On this basis, there are no official monitoring or treatment recommendations for women with AAV who want to become or are pregnant.

Immunomodulatory drugs such as glucocorticoids (GC), azathioprine (AZA), methotrexate (MTX), cyclophosphamide (CYC) and rituximab (RTX) have improved prognosis of AAV in general dramatically [6–9]. More recently, two new treatment options, mepolizumab and avacopan, were approved for EGPA or GPA, respectively [10, 11]. Nevertheless, especially in undiagnosed and thus uncontrolled AAV, mortality due to renal impairment or pulmonary haemorrhages is still high and several of the recommended drugs are incompatible with pregnancy.

This review summarizes the data we have and gives an expert opinion on how to council patients with AAV planning to become pregnant and their management during pregnancy.

## Counselling prior and during pregnancy

Patients with diagnosed AAV planning to become pregnant should be counselled by their rheumatologist and gynaecologist with experiences in high-risk pregnancies, especially concerning potential risk factors, such as disease activity and existing organ damage. Severe organ damage might be an additional risk factor but only rarely a contraindication for pregnancy as long as the vasculitis is truly controlled at the time of conception. Nevertheless, women with severe cardiac and pulmonary involvement with severely reduced ejection fraction or impaired lung function tests due to lung fibrosis or pulmonary hypertension should be counselled against pregnancy (cardiac involvement is present in 6–34% in GPA, < 1% in MPA and 26–54% in EGPA, however, severe cases are rarely observed) [12, 13].

To set optimal conditions organ function should be check and it is strongly recommended that patients are in a sustained remission for at least 6 months on pregnancy compatible medications (see section immunomodulatory drugs in pregnancy). In analogy to pregnancies in connective tissue diseases, we recommend the use of acetylsalicylic-acid to minimize the risk of preeclampsia, even though the benefit in patients with vasculitis has not been investigated in controlled trials. Most frequently acetylsalicylic-acid 100–150 mg/per day until 36 weeks of gestation is used, however, optimal dosing and timing is still a matter of debate [14–16].

## Monitoring disease activity

For monitoring during pregnancy, we recommend a tight control of the patients by clinical rheumatological and gynaecological examinations and laboratory testing every 4–6 weeks, to enable early intervention in case of a disease flare or other complications. Clinical examination must include physical examination including blood pressure measurement. Regularly foetal ultrasound to assess foetal anatomy, growth and placental insufficiency is strongly recommended. Laboratory tests should include blood count and differential blood count (e.g. to detect increasing eosinophilia in EGPA), renal as well as hepatic and inflammatory markers and urine analysis. However, laboratory tests, like C-reactive protein (CRP) and erythrocyte sedimentation rate (ESR) are also influenced by pregnancy and should not automatically be interpreted as a sign of disease activity [17, 18]. The course of ANCA titres might be seen as a marker in ANCA positive patients as flares are often accompanied by increasing ANCA titres, but still its predictive value is rather modest [19]. Urine sediment parameters are of great value in the monitoring of AAV patients, but pregnant women have a non-pathological increase white and red blood cells, epithelial cells and protein, but “active” urine sediment with acanthocytes or casts might be the most reliable parameter to distinguish relapse of renal disease from preeclampsia [20, 21]. Therefore, regular urine analysis with proteinuria and sediment should be performed to detect new or relapsing kidney manifestations. Although imaging procedures are often helpful to diagnose active disease in AAV patients, especially of the sinus and lung, imaging during pregnancy is limited, as both magnetic resonance imaging (MRI) with contrast enhancement and CT should be avoided if possible [22]. Ultrasound is commonly used during pregnancy, its diagnostic value, however, is limited in answering the question of disease activity in small vessel vasculitis. Other common monitoring tools are pulmonary function tests, with its own limitations during pregnancy, as diffusion capacity for carbon monoxide measures should be avoided, although it would be the most sensitive measurement for early interstitial lung disease. In conclusion, disease activity in pregnant women with AAV is evaluated mainly clinically and with selective laboratory testing and thus needs an experienced specialist for AAV.

## Complications and risk factors in pregnancy

Although reported pregnancy outcomes are frequently favourable (with a very limited number of experience), pregnancies in women with AAV should always be considered as high-risk pregnancies. Various complications have been described, from placenta hematoma, intrauterine growth restriction placenta previa, deterioration of organ function to severe maternal and foetal bleeding and death [23]. Complications occur more often in patients with active disease during pregnancy and especially in patients with their first manifestation of AAV during pregnancy, which has been described mainly for GPA and EGPA [24]. For all the data discussed here on complications and their percentages, please keep in mind the low number of reported pregnancies in patients with AAV; the largest cohort so far reported on 106 pregnancies [3]. Table 2 compiles the AAV-associated pregnancy complications.

**Table 2:** Occurrence of pregnancy-associated complications, caveat: all data derived from small patient cohorts

	GPA	MPA	EGPA
Diagnosis in pregnancy	Up to 30%	Rare	30%
Flares	25–40%, higher incidence in AD	Rare, up to 50% in AD	25–50%
Preeclampsia	20%	45%	Rare
IUGR/low body weight	10–25%	Up to 65%	10–30%
Pregnancy loss	5–10%, higher incidences in AD	10%	10–15%, higher incidences in AD
Preterm delivery	50%	N/A	10–40%

AD indicates active disease prior conception

*GPA* granulomatosis with polyangiitis, *MPA* microscopic polyangiitis, *EGPA* eosinophilic granulomatosis with polyangiitis, *IUGR* intrauterine growth restriction

### Preterm birth

Preterm delivery, i.e. birth before 37 weeks of gestation, is the most common pregnancy complication in AAV patients, reported incidences range from 20 to 50% [25–27]. Especially increased vasculitis activity and de novo AAV are associated with preterm delivery, where numbers go as high as 70% [28], whereas in a cohort of 11 women with 15 pregnancies with GPA and MPA in disease-free remission, there was only one preterm birth in a twin pregnancy [29]. Very early delivery (< 34 weeks of gestation) is rarely reported.

### Pregnancy loss

Miscarriage seems to occur more frequently in patients with AAV, with, however, varying incidence rates in the literature between 5 and 20% [4, 26, 28–30]. In a more recent study, Nguyen and colleagues reported on no incidence of miscarriage or stillbirth in their cohort of patients with AAV [25].

### Pregnancy-related high blood pressure disorders

Other frequent complications are preeclampsia and hypertension, which are generally observed more frequently in patients with vasculitis. However, data vary and not all patients are receiving low-dose acetylsalicylic-acid (ASS) prophylaxis, which is suggested to have a protective effect. In the report of Nguyen et al., ASS was used in 6 out of 20 pregnancies at the time of conception with 2/18 cases suffering from preeclampsia [25].

### Flares

Life-threatening exacerbations of AAV occur rarely [25, 28, 30]. Some reports describe maternal deaths due to cardiomyopathy and Corradi et al. reported on a cardiac decompensation which required heart transplantation in close association to delivery [31–33]. As some cases are published decades ago, the outcome might have been better with today’s improved therapeutic options. Nevertheless, more recent case series also describe cases of cardiac or respiratory failure [26, 27]. At least in the report of Pagnoux et al., however, organ involvement was established prior to pregnancy and, especially in the case of cardiac failure, left ventricular function was already impaired before [26].

Taken together, a general elevated risk for cardiac failure in patients with AAV cannot be derived from the current literature, but patients with known cardiac or respiratory involvement, notably patients with EGPA, should be monitored closely.

### ANCA transmission

Up to now, only two cases of diaplacental transmission of MPO-ANCAs with consecutive pulmonary and renal disease of the new born are reported. However, physicians should be aware of this rare but severe complication [34, 35].

## Own experiences

In our own ongoing pregnancy registry for patients with rheumatic diseases, we regarded the most recent cases (*n* = 5 in the last 5 years out of the current 416 pregnancies, we are currently taking care of) of pregnancies in women with AAV (data only partly published) [36]. Their median age was 31 (22–36) years, two suffered from GPA, two from EGPA and one from MPA and only two pregnancies were planned. In one patient who took leflunomide until unexpected conception the foetus suffered from trisomy 18 and the mother decided for interruption. The 4 other patients were on low dose (5–7.5 mg) prednisone at the time of conception and in two pregnancies, there was an ongoing moderate disease activity (recurrent discharge of the ear in a GPA patient, mild dyspnoea in an EGPA patient) and GC dose had to be increased several times and azathioprine was added in one patient. All 4 babies were healthy with normal (median 3475 g) body weight, normal length (median 51 cm) normal pH (median 7.38) and delivered timely (median week 39) and we observed no pregnancy-associated high blood pressure disorders. Taken together, pregnancy outcome was favourable in this few recent cases with frequently monitoring, but ongoing low disease activity occurred twice.

## Immunomodulatory drugs during pregnancy

Several drugs that are used in routine outside pregnancy, namely methotrexate (MTX), leflunomide, mycophenolate mofetil (MMF), as well as cyclophosphamide (CYC) are contraindicated and have to be stopped before conception with adequate distance. If exposed during first trimester, MMF has shown a tenfold increased risk for malformation, whereas with MTX and CYC, the risk is threefold increased [37]. These drugs have to be stopped 3 months before conception and with leflunomide drug elimination procedure with cholestyramine is strongly recommended prior to pregnancy due to its very long half-live [38]. For the new treatment options avacopan [11] and mepolizumab [10], data on pregnancy compatibility are not available and they have to be avoided, too. Mepolizumab, as a monoclonal antibody, does not cross the placental border until week 15; nevertheless, as we aim a stable remission before conception and relapses after discontinuation of mepolizumab often occur, continuation until conception is not yet recommended.

The treating rheumatologist has to decided if the patient is in stable remission and whether immunosuppressive treatment is still needed or if therapy can be stopped; if still needed (e.g. in already existing severe organ damage or in patient with ongoing high titres of ANCAs), it has to be switched in preparation of the pregnancy. In many cases, ongoing immunosuppression is advisable. Treatment options include GCs and azathioprine (AZA), as well as intravenous immunoglobulins (IVIG). In special cases, the immunosuppressive tacrolimus and cyclosporine A can be used if necessary, but both drugs are not in the routine recommendation for AAV treatment as data from studies on their efficacy are missing.

In cases of severe exacerbation, the use of plasma exchange, rituximab, or cyclophosphamide may also be considered in pregnancy:

Although plasma exchange has been shown not to beneficial in AAV patients with acute renal involvement [39], reduction of the autoantibody titres in a life-threatening situation can be helpful and feasible also during pregnancy [28]. Cyclophosphamide is known teratogen, which can cause congenital malformation. In life-threating situations, CYC can be used after the first trimester also in pregnant patients with AAV [38, 40]. Usually, intravenous pulses with 500–750 mg/m^2^ (depending also on renal function) are used. Breastfeeding is contraindicated [40]. Rituximab contains an immunoglobulin G1κ construct and is able to cross the placenta earliest after week 15 of gestation. It depletes B cells in human very effectively and it takes 3–6 months for them to rearise. There is no expected B cell depletion in the newborn, if a woman received rituximab before (4 weeks) conception or during the first trimester [41]. Therefore, rituximab can be used to achieve (usually relatively long lasting) remission in patients with still active disease before conception. In the second or third trimester, however, a profound B cell depletion in both mother and child has to be expected, which normalizes earliest after 3–6 months [42]. The development of the foetal immune system, therefore, is severely influenced with this medication given during the 2 + 3 trimester. EULAR guidelines recommend the use of rituximab in pregnancy only, if there is no other therapeutic option available and in patients who are dependent on this biologic treatment for disease control [40]. Decision should be made by discussing potential risks and benefits through shared decision-making with the patient. Another aspect to consider is the vaccination of the newborn: tremendous B cell depletion after rituximab lowers the response to vaccination and especially live vaccines have to be avoided if exposure has occurred after the 22nd week of pregnancy. Rituximab is compatible with breast feeding and can be re-administered after delivery [40].

## Conclusion

In conclusion, AAV per se is no contraindication for pregnancies but some points have to be considered: in patients with established diagnosis, a stable remission (for 6 months) of disease should be achieved with a medication that can be continued throughout pregnancy. Medication, therefore, has to be adjusted in advance by the treating rheumatologist or an even more specialized centre. In cases of uncontrolled disease flare the potentially fatal risks of the disease has to be respected and necessity of induction therapies with GC + CYC or RTX has to be discussed individually. During pregnancy, a close interdisciplinary monitoring by rheumatologists and gynaecologists and postpartal controls by rheumatologist should be guaranteed. Medications have to be adapted to disease activity.

## References

[1] Kitching AR, Anders HJ, Basu N, Brouwer E, Gordon J, Jayne DR (2020). ANCA-associated vasculitis. Nat Rev Dis Primers.

[2] Mohammad AJ (2020). An update on the epidemiology of ANCA-associated vasculitis. Rheumatology (Oxford).

[3] Singh P, Dhooria A, Rathi M, Agarwal R, Sharma K, Dhir V (2018). Successful treatment outcomes in pregnant patients with ANCA-associated vasculitides: a systematic review of literature. Int J Rheum Dis.

[4] Machen L, Clowse ME (2017). Vasculitis and pregnancy. Rheum Dis Clin North Am.

[5] Golenbiewski JYK, Burroughs C, Kullman J, Merkel P, Clowse M (2019). The vasculitis pregnancy registry (V-PREG): information from the first 3 years arthritis. Rheumatology.

[6] Chanouzas D, McGregor JAG, Nightingale P, Salama AD, Szpirt WM, Basu N (2019). Intravenous pulse methylprednisolone for induction of remission in severe ANCA associated vasculitis: a multi-center retrospective cohort study. BMC Nephrol.

[7] Jain K, Jawa P, Derebail VK, Falk RJ (2021). Treatment Updates in antineutrophil cytoplasmic autoantibodies (ANCA) vasculitis. Kidney360.

[8] Yates M, Watts RA, Bajema IM, Cid MC, Crestani B, Hauser T (2016). EULAR/ERA-EDTA recommendations for the management of ANCA-associated vasculitis. Ann Rheum Dis.

[9] Samman KN, Ross C, Pagnoux C, Makhzoum JP (2021). Update in the management of ANCA-associated vasculitis: recent developments and future perspectives. Int J Rheumatol.

[10] Wechsler ME, Akuthota P, Jayne D, Khoury P, Klion A, Langford CA (2017). Mepolizumab or placebo for eosinophilic granulomatosis with polyangiitis. N Engl J Med.

[11] Jayne DRW, Merkel PA, Schall TJ, Bekker P, Group AS (2021). Avacopan for the treatment of ANCA-associated vasculitis. N Engl J Med.

[12] Misra DP, Shenoy SN (2017). Cardiac involvement in primary systemic vasculitis and potential drug therapies to reduce cardiovascular risk. Rheumatol Int.

[13] Flossmann O, Berden A, de Groot K, Hagen C, Harper L, Heijl C (2011). Long-term patient survival in ANCA-associated vasculitis. Ann Rheum Dis.

[14] Davidson KW, Barry MJ, Mangione CM, Cabana M, Caughey AB, Force USPST (2021). Aspirin use to prevent preeclampsia and related morbidity and mortality: US preventive services task force recommendation statement. JAMA.

[15] Roberge S, Bujold E, Nicolaides KH (2018). Aspirin for the prevention of preterm and term preeclampsia: systematic review and metaanalysis. Am J Obstet Gynecol.

[16] Duley L, Henderson-Smart DJ, Meher S, King JF (2007). Antiplatelet agents for preventing pre-eclampsia and its complications. Cochrane Database Syst Rev.

[17] Watts DH, Krohn MA, Wener MH, Eschenbach DA (1991). C-reactive protein in normal pregnancy. Obstet Gynecol.

[18] van den Broe NR, Letsky EA (2001). Pregnancy and the erythrocyte sedimentation rate. BJOG.

[19] Tomasson G, Grayson PC, Mahr AD, Lavalley M, Merkel PA (2012). Value of ANCA measurements during remission to predict a relapse of ANCA-associated vasculitis–a meta-analysis. Rheumatology (Oxford).

[20] Phillips JK, McBride CA, Hale SA, Solomon RJ, Badger GJ, Bernstein IM (2017). Examination of prepregnancy and pregnancy urinary protein levels in healthy nulliparous women. Reprod Sci.

[21] Lu S, Han M, Wang D (2019). Reference intervals for urine sediment analysis of healthy pregnant women. Clin Lab.

[22] Committee opinion no. 723: guidelines for diagnostic imaging during pregnancy and lactation. Obstet Gynecol. 2017; 130(4):e210–e216. 10.1097/AOG.0000000000002355 10.1097/AOG.0000000000002355 28937575

[23] Gatto M, Iaccarino L, Canova M, Zen M, Nalotto L, Ramonda R (2012). Pregnancy and vasculitis: a systematic review of the literature. Autoimmun Rev.

[24] Ross C, D'Souza R, Pagnoux C (2020). Pregnancy outcomes in systemic vasculitides. Curr Rheumatol Rep.

[25] Nguyen V, Wuebbolt D, Pagnoux C, D'Souza R (2021). Pregnancy outcomes in women with primary systemic vasculitis: a retrospective study. J Matern Fetal Neonatal Med.

[26] Pagnoux C, Le Guern V, Goffinet F, Diot E, Limal N, Pannier E (2011). Pregnancies in systemic necrotizing vasculitides: report on 12 women and their 20 pregnancies. Rheumatology (Oxford).

[27] Fredi M, Lazzaroni MG, Tani C, Ramoni V, Gerosa M, Inverardi F (2015). Systemic vasculitis and pregnancy: a multicenter study on maternal and neonatal outcome of 65 prospectively followed pregnancies. Autoimmun Rev.

[28] Veltri NL, Hladunewich M, Bhasin A, Garland J, Thomson B (2018). De novo antineutrophil cytoplasmic antibody-associated vasculitis in pregnancy: a systematic review on maternal, pregnancy and fetal outcomes. Clin Kidney J.

[29] Croft AP, Smith SW, Carr S, Youssouf S, Salama AD, Burns A (2015). Successful outcome of pregnancy in patients with anti-neutrophil cytoplasm antibody-associated small vessel vasculitis. Kidney Int.

[30] Tuin J, Sanders JS, de Joode AA, Stegeman CA (2012). Pregnancy in women diagnosed with antineutrophil cytoplasmic antibody-associated vasculitis: outcome for the mother and the child. Arthritis Care Res (Hoboken).

[31] Corradi D, Maestri R, Facchetti F (2009). Postpartum Churg-Strauss syndrome with severe cardiac involvement: description of a case and review of the literature. Clin Rheumatol.

[32] Barry C, Davis S, Garrard P, Ferguson IT (1997). Churg-Strauss disease: deterioration in a twin pregnancy. Successful outcome following treatment with corticosteroids and cyclophosphamide. Br J Obstet Gynaecol.

[33] Abul-Haj SK, Flanagan P (1961). Asthma associated with disseminated necrotizing granulomatous vasculitis, the Churg-Strauss syndrome. Report of a case. Med Ann Dist Columbia.

[34] Schlieben DJ, Korbet SM, Kimura RE, Schwartz MM, Lewis EJ (2005). Pulmonary-renal syndrome in a newborn with placental transmission of ANCAs. Am J Kidney Dis.

[35] Bansal PJ, Tobin MC (2004). Neonatal microscopic polyangiitis secondary to transfer of maternal myeloperoxidase-antineutrophil cytoplasmic antibody resulting in neonatal pulmonary hemorrhage and renal involvement. Ann Allergy Asthma Immunol.

[36] Pecher AC, Kagan KO, Wagner M, Abele H, Pauluschke-Froehlich J, Tenev A (2021). Pregnancy outcome is favorable in patients with rheumatic diseases under specialized surveillance - data from the Tuebingen registry for pregnancy in rheumatic diseases in 238 pregnancies. Jt Bone Spine.

[37] Dathe K, Schaefer C (2019). The use of medication in pregnancy. Dtsch Arztebl Int.

[38] Sammaritano LR, Bermas BL, Chakravarty EE, Chambers C, Clowse MEB, Lockshin MD (2020). 2020 American college of rheumatology guideline for the management of reproductive health in rheumatic and musculoskeletal diseases. Arthritis Rheumatol.

[39] Walsh M, Merkel PA, Peh CA, Szpirt WM, Puechal X, Fujimoto S (2020). Plasma exchange and glucocorticoids in severe ANCA-associated vasculitis. N Engl J Med.

[40] Gotestam Skorpen C, Hoeltzenbein M, Tincani A, Fischer-Betz R, Elefant E, Chambers C (2016). The EULAR points to consider for use of antirheumatic drugs before pregnancy, and during pregnancy and lactation. Ann Rheum Dis.

[41] Chakravarty EF, Murray ER, Kelman A, Farmer P (2011). Pregnancy outcomes after maternal exposure to rituximab. Blood.

[42] Schioppo T, Ingegnoli F (2017). Current perspective on rituximab in rheumatic diseases. Drug Des Devel Ther.

